# Randomized, double-blind, placebo-controlled phase I dose escalation study of Dan Qi Tong Mai tablet in healthy volunteers

**DOI:** 10.1186/s12906-019-2751-x

**Published:** 2019-11-27

**Authors:** Zhong-ping Gou, Wei Zhang, Xiu-fang Liang, Ying Wang, Ju-hong Mou, Mei Li, Ya Zhang, Ping Feng

**Affiliations:** 1 Institute of Clinical Trials, West China Hospital, Sichuan University, Chengdu, Sichuan 610041 People’s Republic of China; 2 Cheng Fei Hospital, Chengdu, Sichuan 610041 People’s Republic of China; 3 Taiji Group Research Institute, Chongqing, 401147 People’s Republic of China

**Keywords:** *Salvia miltiorrhiza Bunge*, *Panax notoginseng (Burkill) F.H.Chen*, Dan qi Tong Mai tablet, Phase I dose escalation study, Tolerability

## Abstract

**Background:**

This study aims to assess the tolerability and safety of DQTM tablet, which contains a complex mixture of *Salvia miltiorrhiza* salvianolic acids and *Panax notoginseng* saponins.

**Methods:**

A double-blind, randomized, placebo-controlled phase I dose escalation study was conducted in 84 healthy volunteers. In a single ascending dose study, active ingredients were administered in various doses (90, 270, 540, 1080, 1800, 2880, 4320 or 5760 mg) to 60 subjects in cohorts 1–8. In a multiple ascending dose study, active ingredients were administered at doses of 360, 720 or 2160 mg twice daily to 24 subjects in cohorts 9–11 for 14 consecutive days. Safety was evaluated based on clinical symptoms, vital signs, physical examinations, electrocardiography, laboratory tests and adverse events.

**Results:**

No serious adverse events or clinically significant changes in vital signs or electrocardiography were observed. One subject experienced mildly elevated levels of alanine aminotransferase and aspartate transaminase but recovered spontaneously. Five subjects experienced a small increase in the number of daily stools.

**Conclusions:**

DQTM tablet was well tolerated at single doses of up to 5760 mg and twice-daily doses of up to 2160 mg for 14 consecutive days. The most frequent adverse event was an increase in the number of daily stools.

## Background

Ischemic heart disease is the leading cause of death worldwide [1, 2], and its major type, coronary heart disease, causes an increasing number of deaths each year in China [3]. Approximately 3.5 million people die of cardiovascular disease each year in China, and more than 30% of these deaths are due to ischemic heart disease [4]. Multiple factors contribute to insufficient blood supply to the heart, including critical coronary stenosis, vasospasm, inflammation, microvascular coronary dysfunction, endothelial dysfunction, platelet dysfunction and thrombosis [5]. Several classes of drugs can be used to treat ischemic heart disease: nitroglycerine, statins, angiotensin-converting enzyme inhibitors (ACEI), angiotensin receptor blocker (ARB), calcium channel blockers, β-receptor antagonists, and anti-platelet agents [6, 7]. Certain patients, such as those with left main coronary artery stenosis >50%, may benefit from revascularization, such as percutaneous coronary intervention (PCI) with drug-eluting stents or coronary artery bypass grafting (CABG) [6, 7]. However, revascularization is unsuitable for many patients, such as those with mild angina and no high-risk findings on stress testing, those with simple coronary artery spasm or those with lesions with <50% stenosis. For these patients, medical therapy based on standard guidelines is recommended [6–8]. Furthermore, resistance to nitrates and aspirin can make treatment of ischemic heart disease challenging [9, 10]. Since patients usually require long-term treatment, drug safety remains a significant concern.

Two plants long used in traditional Chinese medicine (TCM) to treat various conditions, including those related to ischemic heart disease, are *Salvia miltiorrhiza Bunge* and *Panax notoginseng (Burkill) F.H.Chen*. They were first described, respectively, in the classics of Chinese Materia Medica *Shen Nong Ben Cao Jin* [11] and *Ben Cao Gang Mu* [12]. They are officially listed in the Chinese Pharmacopeia [13]. *Salvia miltiorrhiza Bunge*, also known as red sage (Danshen), has been used clinically to treat cardiovascular diseases [14–16]. Phenolic acids from this plant, such as salvianolic acids A and B, show anti-platelet and anti-thrombotic activities, which benefit the cardiovascular system [17–27]. *Panax notoginseng* is also known as Sanqi or Tianqi. One of the main active ingredients in *P. notoginseng* is panax notoginseng saponin (PNS), which inhibits inflammation, apoptosis, hypoxia, and coagulation, while promoting angiogenesis [28–32]. PNS contains various chemical components, including noteginsenoside R1, ginsenosides Rg1, Rb1, Re, and Rd. [29, 33–35]. It can be used to treat coronary heart diseases [36–38]. Combination prescriptions such as Fufang Danshen tablet, compound Danshen dripping pill and Danqi tablet are used in the clinic for treatment of coronary heart disease [39–45].

The combination of salvianolic acids and notoginsengnosides has better cardioprotective effects than these components on their own [46, 47]. Dan Qi Tong Mai (DQTM) tablet contains *Salvia miltiorrhiza* salvianolic acids and *Panax notoginseng* saponins, and it shows therapeutic potential against coronary heart disease such as angina pectoris, which falls under the blood stagnation syndrome in traditional Chinese medicine theory. While the ratio of the two components has been optimized in preclinical rat studies in which ligation of the front descending coronary artery was used to mimic acute myocardial infarction, the safety and tolerability of the combination of ingredients have not been reported.

The present study aimed to assess the tolerability and safety of DQTM tablet in healthy volunteers. It also explored preliminary analysis of pharmacodynamics.

## Methods

### Study design

This study was designed as a randomized, double-blind, placebo-controlled, dose escalation clinical trial. The trial was conducted in the Phase I Trial Unit at West China Hospital, Sichuan University (Chengdu, China) after being approved by the Independent Ethics Committee of West China Hospital [2011 Clinical Trial (TCM) Review (No.1)] and the China Food and Drug Administration. Study procedures were conducted in accordance with the Declaration of Helsinki and the principles of the International Conference on Harmonization Guidelines for Good Clinical Practice. The trial was registered at the World Health Organization International Clinical Trial Registry - Chinese Clinical Trial Registry (http://www.chictr.org.cn; registration number: ChiCTR-TRC-12002276). All eligible individuals were informed about the purpose of the trial, study procedures and their risks. Written informed consent was obtained from all subjects participating in the trial.

Table 1 shows the study design and subject allocation. The starting dose was determined based on 1/10 of the projected target therapeutic dose. The estimated maximum dose was calculated to be 1/10 of the maximum dose tolerated in long-term toxicology studies in dogs. Sample size was set based on the literature [48]. In the single ascending dose part of the study, a modified Fibonacci increment strategy was applied, giving rapid escalation in the lower doses and moderate escalation in the higher doses. DQTM tablets were administered to 60 subjects (cohorts 1–8) in the following doses (dose of active ingredients in mg): 90, 270, 540, 1080, 1800, 2880, 4320 or 5760. In the multiple ascending dose part of the study, DQTM tablets were administered twice daily to 24 subjects in cohorts 9–11 for 14 consecutive days in doses of 360, 720 or 2160 mg of active ingredients. The multiple-dose study was performed after the single-dose study. The study began with the lowest-dose group. The next-highest dose was not given until the safety data for the preceding dose had been reviewed.

**Table 1:** Study design and subject allocation

Cohort	Single-dose study (once)								Multiple-dose study (twice daily for 14 days)		
	1	2	3	4	5	6	7	8	9	10	11
Increase,%	Starting dose	200	100	100	67	60	50	33	–	–	–
Dose^a^	90	270	540	1080	1800	2880	4320	5760	360	720	2160
Men	3	4	4	4	4	4	4	3	4	4	4
Women	3	4	4	4	4	4	4	3	4	4	4
Group allocation											
Active ingredients	4	6	6	6	6	6	6	4	6	6	6
Placebo	2	2	2	2	2	2	2	2	2	2	2
Total	6	8	8	8	8	8	8	6	8	8	8

^a^mg of active ingredients

Volunteers were instructed to abstain from taking any drugs, including over-the counter medications, for 2 weeks before and during the study period. They were also asked to abstain from alcohol, smoking, intense physical activity, and caffeine-containing beverages during the study.

### Inclusion and exclusion criteria

Healthy men and women between 18 and 45 years old who reported being non-smokers and had a body mass index between 19 and 24 were eligible for recruitment. Subjects had to be healthy based on medical history and on the following tests conducted within 2 weeks before the first drug dose: physical examination, 12-lead electrocardiography, clinical laboratory tests (hematology, blood chemistry, urinalysis), and serology (hepatitis B surface antigen, hepatitis C virus antibody, and HIV antigen/antibody).

Subjects were excluded from the study if they had any allergies or history of cardiac, pulmonary, renal, hepatic, gastrointestinal, or hematologic abnormality or any other acute or chronic disease. Subjects were also excluded if they had received any other investigational medication within 30 days of the first dose of study medication, if they were pregnant or nursing, or if they were women with childbearing potential who did not use a highly effective birth control method.

### Study medication

DQTM tablet was manufactured from farmed plant material by Chongqing Fuling Pharmaceutical of the Taiji Group (Chongqing, China) in two proprietary formulations: dosage I (batch number: 10110001) contained 180 mg of salvianolic acids and saponins (active ingredients), and dosage II (batch number: 10110002) contained 90 mg of these ingredients. Plant species were identified based on the criteria of the Chinese Pharmacopoeia Commission [49]. The major active ingredients included Salvianolic acid B, Ginsenoside Rg1, Ginsenoside Rb1 and Panax notoginseng saponins R1. The final formulation was prepared using ethanol precipitation, macroreticular adsorbent and reducing pressure drying. The placebo was also manufactured by Chongqing Fuling Pharmaceutical.

### Randomization and blinding

Randomization was carried out by a biostatistician using the Proc Plan Procedure in SAS 9.2 (SAS Institute, Cary, North Carolina, USA). Subjects were stratified by sex (1, 1 ratio). In cohorts 1 and 8, subjects were randomly allocated in a 2:1 ratio to receive active ingredients or placebo; in cohorts 2–7 and 9–11, the allocation ratio was 3:1 (Table 1). The randomization lists for each group were placed in sealed envelopes, and the details remained unknown to the study investigators. While researchers and subjects knew the allocated dose, they were blinded to whether the subject received drug or placebo.

### Assessments

Compliance and adherence to treatment were assessed based on the ratio of actual dosage to planned dosage for each subject. Vital signs were measured at screening, pre-dose, and at 2, 4, 8 and 24 h post-dose in the single-dose study. Vital signs were measured at screening, each drug administration day (Days 1–14) and Day 15 in the multiple-dose study. Physical examination, 12-lead electrocardiography and laboratory tests (hematology, blood chemistry, blood coagulation, urinalysis) were conducted at screening and at the end of the study. Laboratory tests and 12-lead electrocardiography were also performed on Day 8 in the multiple-dose study. Haematology tests included red blood cell count, hemoglobin, white blood cell count, percentage of neutrophils, percentage of lymphocytes, and percentage of monocytes. Blood chemistry included total bilirubin, direct bilirubin, indirect bilirubin, alanine aminotransferase (ALT), aspartate aminotransferase (AST), blood urea nitrogen (BUN), creatinine, blood glucose, triglyceride, cholesterol, high-density lipoprotein, low-density lipoprotein, potassium, sodium, and chloride. All laboratory tests were performed in the clinical laboratory of West China Hospital, Sichuan University, which is accredited by the College of American Pathologists.

Tolerance and safety were evaluated based on clinical symptoms, vital signs, physical examinations, electrocardiography, laboratory tests and adverse events. Adverse events were recorded on a prespecified form. We chose not to give subjects a preformulated questionnaire about adverse events in order to avoid inducement effects linked to over-reporting. Adverse events were classified as *related, probably related, possibly related, possibly unrelated,* or *unrelated* to the study medication. Adverse drug reactions were adverse events judged to be *related, probably related* or *possibly related* to the study medication.

In the multiple-dose part of the study, blood coagulation and adenosine diphosphate (ADP)-induced platelet aggregation were tested at baseline and on Days 8 and 15 after the first dose. The following blood coagulation parameters were measured: prothrombin time (PT), activated partial thromboplastin time (APTT), thrombin time (TT), international normalized ratio (INR) and fibrinogen.

### Statistical analyses

All analyses were performed using SAS 9.2 software with a significance level of 0.05. All analyses were based on an intention-to-treat approach. Inter-group differences in continuous variables were assessed for significance using a group *t* test or Wilcoxon rank test, while differences in categorical variables were assessed using chi-squared and Fisher’s exact tests. Subjects in the placebo group in each cohort were pooled into a single control group in the single- and multiple-dose studies. This procedure is aligned with practices in the literature [50, 51]. In the multiple-dose part of the study, repeated-measures analysis of variance was used to assess the significance of changes in variables related to platelet aggregation and coagulation.

## Results

**Fig. 1:**
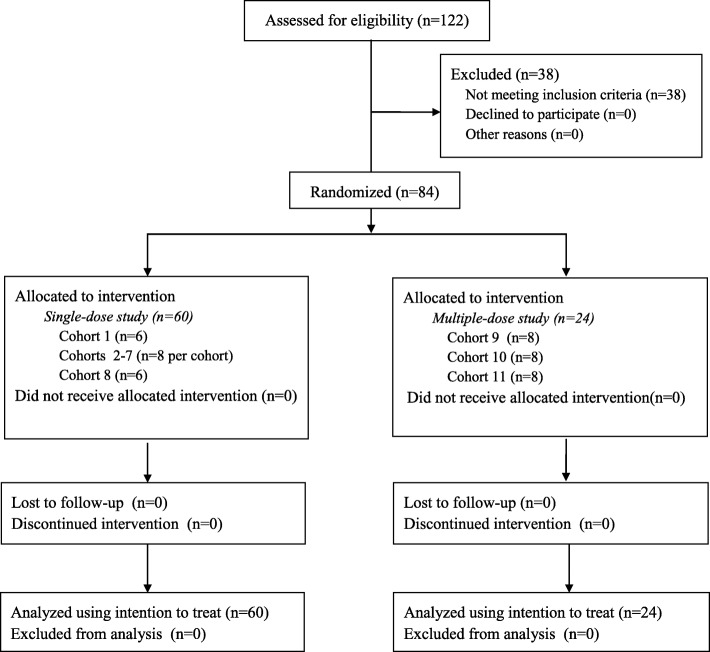
Flowchart of the study procedure

A total of 122 healthy volunteers were screened, and 84 underwent random allocation and completed the study (Fig. 1). All drugs were administered by trained researchers in the Phase I Trial Unit, and subject compliance with treatment was 100%. All enrolled subjects were included in tolerability assessment. The single-dose part of the study contained 60 subjects; the multiple-dose part, 24 subjects (Table 2). Subjects receiving active ingredients or placebo did not differ significantly in age or body mass index.

**Table 2:** Characteristics of subjects in each cohort

Parameter	Study	Cohort	Group receiving active ingredients	Group receiving placebo	Z*	P*
Age (year)	Single-dose	1	29.00 (9.59)	24.00 (7.07)	−0.23	0.83
		2	21.83 (1.47)	22.00 (0.00)	0.17	0.87
		3	23.83 (1.17)	22.00 (1.41)	−0.69	0.51
		4	24.00 (2.53)	24.50 (3.54)	0.00	1.00
		5	23.83 (2.04)	23.00 (1.41)	−0.34	0.75
		6	23.83 (0.98)	23.00 (1.41)	0.00	1.00
		7	25.67 (1.97)	25.00 (0.00)	−0.17	0.87
		8	24.75 (0.96)	24.00 (1.41)	−0.48	0.65
	Multiple-dose	9	24.00 (2.28)	24.00 (1.41)	0.17	0.87
		10	23.83 (2.40)	23.50 (3.54)	0.00	1.00
		11	22.50 (1.64)	22.50 (0.71)	1.70	0.13
Height (cm)	Single-dose	1	167.00 (8.08)	168.50 (12.02)	0.00	1.00
		2	165.17 (9.85)	162.50 (10.61)	−0.34	0.74
		3	169.17 (7.08)	169.00 (15.56)	0.00	1.00
		4	167.67 (6.98)	166.00 (8.45)	0.00	1.00
		5	168.17 (4.71)	166.00 (8.49)	−0.17	0.87
		6	165.17 (12.06)	164.50 (7.78)	0.17	0.87
		7	166.33 (6.95)	167.00 (14.14)	0.00	1.00
		8	166.50 (6.95)	160.00 (0.00)	−0.70	0.48
	Multiple-dose	9	163.00 (9.12)	164.00 (5.66)	0.34	0.74
		10	169.50 (7.45)	172.50 (0.71)	0.50	0.62
		11	167.00 (7.32)	159.00 (12.73)	−0.67	0.50
Weight (kg)	Single-dose	1	57.75 (9.22)	62.00 (14.14)	0.46	0.64
		2	56.33 (10.54)	58.00 (4.24)	0.50	0.62
		3	58.83 (10.63)	64.00 (12.73)	0.67	0.50
		4	60.83 (8.80)	58.50 (9.19)	−0.17	0.86
		5	59.83 (4.31)	54.50 (7.78)	−1.07	0.29
		6	58.17 (9.06)	56.50 (10.61)	−0.51	0.61
		7	59.00 (7.32)	56.50 (13.44)	−0.17	0.87
		8	59.50 (6.66)	49.50 (0.71)	−1.41	0.16
	Multiple-dose	9	58.17 (12.95)	57.50 (3.54)	0.00	1.00
		10	62.33 (9.35)	62.50 (3.54)	0.17	0.87
		11	60.42 (8.18)	54.75 (13.79)	−0.50	0.62
Body mass index (kg/m^2^)	Single-dose	1	20.59 (1.23)	21.60 (1.83)	1.16	0.25
		2	20.53 (1.63)	22.00 (1.26)	0.83	0.40
		3	20.41 (2.03)	22.28 (0.35)	1.17	0.24
		4	21.54 (1.73)	21.14 (1.17)	−0.17	0.87
		5	21.14 (1.00)	19.72 (0.82)	−1.50	0.13
		6	21.25 (1.47)	20.77 (1.96)	−0.50	0.62
		7	21.30 (2.08)	20.07 (1.41)	−0.83	0.40
		8	21.40 (0.95)	19.34 (0.28)	−1.62	0.11
	Multiple-dose	9	21.24 (1.67)	21.37 (0.16)	0.00	1.00
		10	21.58 (1.52)	21.01 (1.36)	−0.84	0.40
		11	21.57 (1.39)	21.43 (2.02)	0.00	1.00

Values are mean (SD)

^*^Wilcoxon test

After drug administration, none of the cohorts showed clinically or statistically significant variations in vital signs, hematology, blood chemistry, urinalysis or electrocardiography (Additional file 1: Table S1). Treatment and placebo groups differed significantly in changes from baseline in the following cases: cohort 1 for TT; cohort 3 for PT and INR; cohort 4 for red blood cell count, hemoglobin, platelet count, total bilirubin, PT, and INR; and cohort 7 for creatinine. However, none of these differences was clinically significant.

Table 3 summarizes adverse events in the study. Six of 72 (8.3%) subjects who received active ingredients experienced adverse events and adverse drug reactions. In the single-dose part of the study, four subjects who received active ingredients experienced adverse events, which were classified as *related* to the study drug: one subject in cohort 4 and two in cohort 8 experienced one or two additional stools per day over baseline. Stool analyses were normal, and fecal occult blood tests were negative. One subject in cohort 4 experienced mildly elevated ALT and AST values, which self-resolved. No adverse event was observed in the placebo group. In the multiple-dose part of the study, two subjects who received active ingredients experienced mild adverse events that were judged to be *related* to the study drug: one subject in cohort 11 experienced one or two additional stools per day over baseline for 3 days, and one subject in cohort 11 experienced two additional stools over baseline during 1 day of drug administration. Stool analyses were normal and fecal occult blood tests were negative. One subject in the placebo group in cohort 11 experienced cough and runny nose, which was mild and judged to be *unrelated* to the study drug. No intervention was taken against the adverse events, and no subject discontinued the study because of such events.

**Table 3:** Summary of adverse events

Parameter	Adverse event		
	Increase in no. daily stools	Elevated ALT and AST	Cough and runny nose
No. cases	5	1	1
Cohort (no. of cases)	4 (1), 8 (2), 11 (2)	4 (1)	11 (1)
Group	Active ingredients	Active ingredients	Placebo
Severity	Mild	Mild	Mild
Duration, days	1–2	5	16
Relationship to study drug	Probably or possibly related	Probably related	Unrelated
Outcome	Recovered spontaneously	Recovered spontaneously	Recovered spontaneously

*ALT* Alanine aminotransferase, *AST* Aspartate transaminase

Table 4 presents repeated-measures analysis of variance in platelet aggregation in the multiple-dose part of the study. Platelet aggregation did not change significantly over time within each group, nor did it differ significantly between groups over time. Groups receiving active ingredients or placebo did not differ significantly in platelet aggregation, PT, APTT, TT, INR or fibrinogen (data not shown).

**Table 4:** Platelet aggregation (%) at different time points

Treatment	Cohort	Dose, mg twice daily	Baseline	Day 8	Day 15
Placebo	–	–	61.50 (24.34)	52.67 (23.84)	68.17 (13.82)
Active ingredients	9	360	56.17 (31.56)	68.83 (13.24)	55.00 (15.19)
Active ingredients	10	720	63.50 (14.17)	42.83 (13.91)	58.00 (17.78)
Active ingredients	11	2160	77.00 (7.24)	67.00 (16.78)	72.83 (12.73)
Repeated-measures analysis of variance	Group	F	2.83		
		*P* value	0.06		
	Time	F	0.97		
		P value	0.36		
	Group*Time	F	1.29		
		*P* value	0.3		

Values are mean (SD), unless otherwise noted

*Denote the interaction

## Discussion

Like many traditional Chinese medicines, the combination of *Salvia miltiorrhiza* and *Panax notoginseng* has been used clinically for a long time, yet its formulation in the DQTM tablet appears not to have been rigorously assessed for safety and tolerability. Here we conducted such an assessment by administering single and multiple doses of DQTM tablet to 84 healthy Chinese volunteers.

All volunteers who enrolled in our study completed it, and compliance with treatment was 100%. We did not observe any clinically or statistically significant changes in vital signs, hematology, blood chemistry, urinalysis or electrocardiography at any dose tested, including at a single dose of 5760 mg, which is approximately 10 times the dose routinely used in the clinic. These results suggest that DQTM is well tolerated.

Given the small sample in this phase I trial, we analyzed individual-level data. We observed that the most frequent adverse event after study drug administration was an increase in the number of daily stools. These events occurred primarily in the single-dose groups receiving intermediate and high doses, as well as in the multiple-dose group receiving the highest dose. All these events, which were classified as *related* to DQTM, were mild. These results suggest that DQTM may stimulate peristalsis of the gastrointestinal tract, which may be a beneficial side effect for ischemic heart disease patients with constipation. At the same time, one subject in cohort 4 who received a single dose of 1080 mg experienced mildly elevated ALT and AST. This suggests the potential of DQTM to cause clinically important adverse drug reactions, which should be monitored in future work.

The mechanism of action of DQTM tablet is not completely clear. *Salvia miltiorrhiza* and *Panax notoginseng* exhibit various bioactivities related to processes underlying ischemic heart disease [23, 29]: they have been shown to improve lipid metabolism in an ischemic heart model in rats [52, 53], improve heart contractility in rats with myocardial infarction [54], and exert cardioprotection against ischemia/reperfusion injury in rats [55]. We focused on coagulation function and platelet aggregation in this phase I study because the two plants have been shown to inhibit platelet aggregation or affect coagulation in animal studies [17–19, 24, 25, 31, 47], patients with hypertension [16], and patients with acute coronary syndrome [22]. Preliminary pharmacodynamics analysis in the multiple-dose part of our study did not suggest an effect of DQTM on coagulation or platelet aggregation. However, we did observe a trend (*P* = 0.06) toward platelet aggregation on Day 8 in subjects receiving 720 or 2160 mg. Our failure to observe significant anti-platelet or anti-coagulation effects may reflect our small sample, especially since baseline platelet aggregation varied substantially. It may also reflect the fact that our study involved healthy individuals rather than patients.

Although we chose our sample based on the literature [48], the small size may limit the reliability of our findings. Large studies in patients are needed to explore the mechanism of DQTM tablet. Future work should aim to identify individual active ingredients in the complex mixture of the DQTM tablet. It should also analyze DQTM pharmacokinetics in detail, which is a bottleneck for many traditional Chinese medicines.

## Conclusion

This study suggests that DQTM tablet is well tolerated at single doses of up to 5760 mg and twice-daily doses of up to 2160 mg for 14 consecutive days. The most frequent adverse event was an increase in the number of daily stools. Based on these encouraging safety results, a randomized, placebo-controlled, multi-center phase II study has been launched to evaluate the efficacy of DQTM tablet for ischemic heart disease.

## Supplementary information

**Additional file 1: Table S1.** Changes in hematology, blood chemistry and coagulation from baseline in treatment and placebo groups.

## Data Availability

Data are available from the corresponding author upon reasonable request and the approval of the data owner.

## Abbreviations

ACEI: Angiotensin-converting enzyme inhibitors
ADP: Adenosine diphosphate
ALT: Alanine aminotransferase
APTT: Activated partial thromboplastin time
ARB: Angiotensin receptor blocker
AST: Aspartate transaminase
CABG: Coronary artery bypass grafting
DQTM: Dan Qi Tong Mai
INR: International normalized ratio
PCI: Percutaneous coronary intervention
PT: Prothrombin time
SAS: Statistical Analysis System
SD: Standard deviation
TT: Thrombin time
