# Supplementary material for: Randomized, double-blind, placebo-controlled phase I dose escalation study of Dan Qi Tong Mai tablet in healthy volunteers
Source: BMC Complement Altern Med. 2019 Nov 27;19:336. doi: 10.1186/s12906-019-2751-x (PMC6882005; doi:10.1186/s12906-019-2751-x)
Supplement: Supplementary file 1 — Additional file 1: Table S1. Changes in hematology, blood chemistry and coagulation from baseline in treatment and placebo groups. [file 12906_2019_2751_MOESM1_ESM.doc]

Supplementary Material

Table 1 Changes in hematology, blood chemistry and coagulation from baseline in treatment and placebo groups

| Parameter | Group | Cohort | Baseline | After test | Z* | *P value* |
| --- | --- | --- | --- | --- | --- | --- |
| Red blood cells (10^12^/L) | Placebo | - | 4.70 (0.48) | 4.71 (0.46) | - | - |
|  | Single-dose | 1 | 5.05 (0.75) | 4.97 (0.67) | -0.47 | 0.64 |
|  |  | 2 | 4.75 (0.51) | 4.70 (0.56) | -0.81 | 0.43 |
|  |  | 3 | 4.83 (0.53) | 4.77 (0.54) | -1.29 | 0.21 |
|  |  | 4 | 4.65 (0.60) | 4.49 (0.57) | -2.40 | 0.03 |
|  |  | 5 | 4.76 (0.44) | 4.85 (0.53) | 0.77 | 0.45 |
|  |  | 6 | 4.87 (0.45) | 4.76 (0.53) | -1.73 | 0.10 |
|  |  | 7 | 4.57 (0.51) | 4.66 (0.48) | 0.96 | 0.35 |
|  |  | 8 | 4.95 (0.65) | 5.04 (0.59) | 1.18 | 0.25 |
|  | Placebo | - | 4.65 (0.55) | 4.68 (0.51) | - | - |
|  | Multiple-dose | 9 | 4.67 (0.47) | 4.59 (0.36) | -0.40 | 0.70 |
|  |  | 10 | 4.91 (0.62) | 4.82 (0.62) | -0.24 | 0.81 |
|  |  | 11 | 4.78 (0.40) | 4.58 (0.35) | -0.80 | 0.44 |
| Hemoglobin (g/L) | Placebo | - | 141.81 (14.36) | 143.19 (13.79) | - | - |
|  | Single-dose | 1 | 147.00 (22.52) | 145.50 (20.53) | -0.72 | 0.48 |
|  |  | 2 | 145.17 (18.48) | 142.50 (19.66) | -1.71 | 0.10 |
|  |  | 3 | 138.17 (15.50) | 137.50 (14.82) | -1.31 | 0.20 |
|  |  | 4 | 139.17 (17.05) | 134.17 (16.17) | -3.46 | 0.00 |
|  |  | 5 | 142.50 (14.69) | 144.33 (18.03) | 0.52 | 0.61 |
|  |  | 6 | 143.00 (12.46) | 141.17 (14.39) | -1.63 | 0.12 |
|  |  | 7 | 136.67 (15.24) | 138.50 (15.25) | 0.11 | 0.91 |
|  |  | 8 | 146.25 (19.72) | 149.75 (17.73) | 1.15 | 0.27 |
|  | Placebo | - | 140.33 (20.50) | 140.33 (20.75) | - | - |
|  | Multiple-dose | 9 | 141.17 (19.47) | 138.50 (13.69) | -0.24 | 0.81 |
|  |  | 10 | 145.00 (14.24) | 141.33 (14.75) | -0.48 | 0.64 |
|  |  | 11 | 142.67 (11.36) | 136.17 (9.28) | 0.40 | 0.70 |
| Platelets (10^9^/L) | Placebo | - | 204.69(38.77) | 215.81(34.56) | - | - |
|  | Single-dose | 1 | 171.25 (38.77) | 174.00 (36.62) | -0.95 | 0.36 |
|  |  | 2 | 165.50 (24.45) | 183.83 (28.21) | 1.22 | 0.24 |
|  |  | 3 | 205.17 (47.11) | 214.83 (51.79) | 0.04 | 0.97 |
|  |  | 4 | 230.50 (51.35) | 268.00 (37.98) | 2.10 | 0.05 |
|  |  | 5 | 264.83 (70.37) | 267.83 (61.61) | -0.70 | 0.49 |
|  |  | 6 | 210.83 (50.84) | 219.33 (37.55) | -0.33 | 0.74 |
|  |  | 7 | 235.50 (45.48） | 258.67 (54.51) | 0.85 | 0.41 |
|  |  | 8 | 209.00 (39.35) | 234.75 (18.28) | 0.66 | 0.52 |
|  | Placebo | - | 234.83 (49.00) | 234.17 (63.95) | - | - |
|  | Multiple-dose | 9 | 207.67 (66.28) | 201.50 (31.65) | 0.56 | 0.59 |
|  |  | 10 | 235.50 (52.84) | 217.00 (31.27) | 0.40 | 0.70 |
|  |  | 11 | 240.50 (37.91) | 247.50 (51.99) | -0.08 | 0.94 |
| White blood cells (10^9^/L) | Placebo | - | 5.29 (1.02) | 5.60 (0.80) | - | - |
|  | Single-dose | 1 | 5.75 (1.39) | 5.20 (0.94) | -0.99 | 0.33 |
|  |  | 2 | 5.07 (0.82) | 5.20 (0.94) | -1.07 | 0.30 |
|  |  | 3 | 5.91 (1.43) | 5.85 (1.07) | -1.29 | 0.20 |
|  |  | 4 | 5.45 (0.78) | 5.28 (0.85) | -1.88 | 0.07 |
|  |  | 5 | 5.95 (1.60) | 5.63 (0.65) | -0.63 | 0.54 |
|  |  | 6 | 5.76 (1.16) | 6.12 (0.76) | -0.11 | 0.91 |
|  |  | 7 | 5.81 (1.50) | 5.68 (1.58) | -1.00 | 0.33 |
|  |  | 8 | 5.09 (0.77) | 5.46 (0.54) | 0.14 | 0.89 |
|  | Placebo | - | 5.12 (0.74) | 5.10 (0.84) | - | - |
|  | Multiple-dose | 9 | 5.45 (1.41) | 5.72 (0.63) | 0.40 | 0.70 |
|  |  | 10 | 5.30 (0.62) | 5.08 (0.77) | -0.08 | 0.94 |
|  |  | 11 | 6.06 (1.04) | 5.61 (0.71) | -0.88 | 0.40 |
| Total bilirubin (μmol/L) | Placebo | - | 13.47 (3.96) | 11.55 (4.69) | - | - |
|  | Single-dose | 1 | 15.05 (4.74) | 14.78 (3.47) | 0.90 | 0.38 |
|  |  | 2 | 16.75 (2.24) | 12.03 (3.53) | -1.59 | 0.13 |
|  |  | 3 | 13.40 (2.13) | 12.20 (4.98) | 0.26 | 0.80 |
|  |  | 4 | 12.48 (4.95) | 14.62 (5.05) | 2.06 | 0.05 |
|  |  | 5 | 12.93 (4.45) | 12.68 (4.53) | 0.55 | 0.59 |
|  |  | 6 | 13.18 (2.93) | 10.20 (3.69) | -0.77 | 0.45 |
|  |  | 7 | 10.62 (2.28) | 13.62 (7.42) | 1.77 | 0.09 |
|  |  | 8 | 11.88 (3.84) | 15.40 (5.93) | 1.09 | 0.29 |
|  | Placebo | - | 13.25 (4.95) | 15.37 (10.20) | - | - |
|  | Multiple-dose | 9 | 13.90 (7.21) | 16.12 (5.08) | 0.88 | 0.40 |
|  |  | 10 | 12.07 (3.29) | 15.17 (2.33) | -0.24 | 0.81 |
|  |  | 11 | 11.72 (6.16) | 12.52 (3.45) | 0.72 | 0.49 |
| Alanine aminotransferase (IU/L) | Placebo | - | 20.94 (10.86) | 20.81 (11.37) | - | - |
|  | Single-dose | 1 | 25.50 (18.12) | 21.75 (16.13) | -1.00 | 0.33 |
|  |  | 2 | 18.17 (6.74) | 16.50 (5.13) | 0.00 | 1.00 |
|  |  | 3 | 14.00 (6.39) | 14.00 (4.86) | 0.37 | 0.72 |
|  |  | 4 | 23.00 (16.85) | 29.17 (21.92) | 0.74 | 0.47 |
|  |  | 5 | 14.33 (6.74) | 12.83 (2.32) | 0.00 | 1.00 |
|  |  | 6 | 18.17 (6.46) | 14.67 (7.12) | -1.26 | 0.22 |
|  |  | 7 | 14.00 (2.97) | 11.67 (2.42) | -0.41 | 0.69 |
|  |  | 8 | 19.75 (11.32) | 23.00 (11.69) | 1.18 | 0.25 |
|  | Placebo | - | 16.17 (6.62) | 16.33 (6.65) | - | - |
|  | Multiple-dose | 9 | 14.83 (7.28) | 17.50 (5.54) | -0.24 | 0.81 |
|  |  | 10 | 16.17 (10.26) | 14.50 (4.46) | 0.18 | 0.86 |
|  |  | 11 | 17.83 (7.70) | 14.17 (2.64) | -0.33 | 0.74 |
| Aspartate aminotransferase (IU/L) | Placebo | - | 22.69 (4.78) | 22.13 (6.45) | - | - |
|  | Single-dose | 1 | 27.00 (14.02) | 21.25 (10.90) | -1.72 | 0.10 |
|  |  | 2 | 21.17 (3.60) | 20.67 (2.73) | 0.04 | 0.97 |
|  |  | 3 | 19.67 (4.50) | 20.50 (6.60) | 0.26 | 0.80 |
|  |  | 4 | 22.00 (5.69) | 25.67 (12.21) | 0.56 | 0.58 |
|  |  | 5 | 18.17 (1.60) | 16.50 (2.35) | -0.52 | 0.61 |
|  |  | 6 | 21.00 (3.16) | 20.00 (3.35) | 0.00 | 1.00 |
|  |  | 7 | 18.67 (1.86） | 17.67 (2.58) | 0.00 | 1.00 |
|  |  | 8 | 21.75 (7.50) | 22.25 (6.18) | 0.76 | 0.46 |
|  | Placebo | - | 18.33 (3.08) | 18.83 (4.26) | - | - |
|  | Multiple-dose | 9 | 22.67 (7.31) | 20.33 (4.50) | -1.94 | 0.08 |
|  |  | 10 | 19.83 (3.43) | 18.50 (2.43) | -1.13 | 0.28 |
|  |  | 11 | 19.83 (7.94) | 16.50 (4.28) | -0.17 | 0.87 |
| Creatinine (μmol/L) | Placebo | - | 71.66 (15.17) | 68.86 (13.02) | - | - |
|  | Single-dose | 1 | 59.25 (8.22) | 62.73 (4.12) | 1.47 | 0.16 |
|  |  | 2 | 74.27 (17.20) | 68.73 (15.03) | -0.74 | 0.47 |
|  |  | 3 | 66.25 (11.68) | 65.98 (10.41) | 1.07 | 0.30 |
|  |  | 4 | 66.45 (11.01) | 63.93 (8.67) | 0.00 | 1.00 |
|  |  | 5 | 72.92 (13.26) | 69.28 (14.11) | -0.11 | 0.91 |
|  |  | 6 | 77.25 (4.95) | 73.72 (9.76) | -0.26 | 0.80 |
|  |  | 7 | 67.35 (9.94) | 72.18 (11.98) | 2.32 | 0.03 |
|  |  | 8 | 68.60 (14.67) | 72.23 (13.60) | 1.84 | 0.08 |
|  | Placebo | - | 76.60 (14.20) | 79.80 (16.82) | - | - |
|  | Multiple-dose | 9 | 75.98 (11.57) | 69.82 (13.75) | -1.52 | 0.16 |
|  |  | 10 | 71.52 (18.75) | 71.12 (15.85) | -0.24 | 0.81 |
|  |  | 11 | 71.57 (18.98) | 71.33 (15.29) | 0.48 | 0.64 |
| Triglyceride (mmol/L) | Placebo | - | 0.82 (0.36) | 0.93 (0.42) | - | - |
|  | Single-dose | 1 | 0.84 (0.11) | 1.01 (0.32) | 0.71 | 0.49 |
|  |  | 2 | 0.94 (0.24) | 0.78 (0.21) | -1.44 | 0.17 |
|  |  | 3 | 0.73 (0.15) | 0.80 (0.09) | 0.37 | 0.72 |
|  |  | 4 | 1.15 (0.53) | 0.99 (0.39) | -1.22 | 0.24 |
|  |  | 5 | 1.06 (0.48) | 1.29 (0.50) | 1.22 | 0.24 |
|  |  | 6 | 0.93 (0.28) | 1.02 (0.28) | 0.48 | 0.64 |
|  |  | 7 | 0.75 (0.20) | 0.97 (0.38) | 1.22 | 0.24 |
|  |  | 8 | 0.71 (0.18) | 0.85 (0.15) | 0.43 | 0.68 |
|  | Placebo | - | 0.82 (0.23) | 0.80 (0.47) | - | - |
|  | Multiple-dose | 9 | 0.89 (0.33) | 0.68 (0.22) | -0.88 | 0.40 |
|  |  | 10 | 0.64 (0.14) | 0.68 (0.25) | 1.20 | 0.25 |
|  |  | 11 | 0.75 (0.15) | 0.64 (0.18) | 0.24 | 0.81 |
| Cholesterol (mmol/L) | Placebo | - | 3.87 (0.74) | 3.98 (0.67) | - | - |
|  | Single-dose | 1 | 4.19 (0.63) | 4.31 (0.81) | -0.14 | 0.89 |
|  |  | 2 | 3.83 (0.82) | 3.87 (0.57) | -0.52 | 0.61 |
|  |  | 3 | 4.17 (0.22) | 4.24 (0.40) | -0.18 | 0.86 |
|  |  | 4 | 4.10 (0.70) | 4.42 (0.48) | 1.44 | 0.17 |
|  |  | 5 | 4.60 (0.80) | 4.76 (0.71) | 0.00 | 1.00 |
|  |  | 6 | 4.24 (0.68) | 4.22 (0.71) | -1.03 | 0.31 |
|  |  | 7 | 4.32 (0.91) | 4.43 (0.84) | -0.18 | 0.86 |
|  |  | 8 | 4.12 (0.48) | 4.36 (0.42) | 0.90 | 0.38 |
|  | Placebo | - | 4.16 (0.67) | 4.44 (0.76) | - | - |
|  | Multiple-dose | 9 | 3.76 (0.38) | 3.70 (0.64) | -0.72 | 0.47 |
|  |  | 10 | 3.50 (0.45) | 3.92 (0.36) | 0.40 | 0.70 |
|  |  | 11 | 4.03 (0.41) | 3.83 (0.43) | -1.04 | 0.32 |
| Plasma prothrombin time (s) | Placebo | - | 11.10 (0.66) | 11.05 (0.74) | - | - |
|  | Single-dose | 1 | 10.58 (0.37) | 10.93 (0.60) | 1.47 | 0.16 |
|  |  | 2 | 11.33 (0.43) | 10.85 (0.61) | -0.26 | 0.80 |
|  |  | 3 | 11.28 (0.40) | 10.85 (0.61) | -2.10 | 0.05 |
|  |  | 4 | 10.80 (0.52) | 11.30 (0.28) | 2.14 | 0.04 |
|  |  | 5 | 11.08 (0.36) | 12.03 (1.27) | 1.88 | 0.06 |
|  |  | 6 | 10.90 (0.37) | 10.98 (0.58) | 0.41 | 0.68 |
|  |  | 7 | 10.85 (0.42) | 11.45 (0.63) | 2.00 | 0.05 |
|  |  | 8 | 11.10 (0.14) | 11.15 (0.45) | 0.24 | 0.82 |
|  | Placebo | - | 11.65 (0.73) | 11.40 (0.67) | - | - |
|  | Multiple-dose | 9 | 11.22 (0.75) | 11.43 (0.83) | -0.40 | 0.70 |
|  |  | 10 | 11.73 (0.37) | 11.48 (0.48) | 0.48 | 0.64 |
|  |  | 11 | 11.07 (0.63) | 11.35 (0.36) | 1.29 | 0.23 |
| [International normalized ratio](http://www.baidu.com/link?url=S7luyQDIYM-w9dYzgiihocRd6B0FoN3dLGz-RPAQUr94O8tlIa4fgbVk_TWPbqUfdMygk0BNzz29byKEN8DP8N9Vriic76EmRd8JjxJpIB2G74eIFhm0bPyADfIqd8DV4OtzdvlH7-0x1_zM_yIp5a) | Placebo | - | 1.00 (0.06) | 0.99 (0.07) | - | - |
|  | Single-dose | 1 | 0.95 (0.04) | 0.98 (0.06) | 1.61 | 0.12 |
|  |  | 2 | 1.02 (0.04) | 0.98 (0.06) | -0.19 | 0.86 |
|  |  | 3 | 1.02 (0.04) | 0.98 (0.06) | -2.11 | 0.05 |
|  |  | 4 | 0.97 (0.05) | 1.02 (0.03) | 2.29 | 0.03 |
|  |  | 5 | 1.00 (0.04) | 1.09 (0.12) | 1.92 | 0.07 |
|  |  | 6 | 0.98 (0.04) | 0.99 (0.06) | 0.48 | 0.63 |
|  |  | 7 | 0.98 (0.04) | 1.02 (0.06) | 1.78 | 0.09 |
|  |  | 8 | 0.99 (0.01) | 1.00 (0.04) | 0.29 | 0.78 |
|  | Placebo | - | 1.04 (0.07) | 1.02 (0.06) | - | - |
|  | Multiple-dose | 9 | 1.00 (0.07) | 1.02 (0.07) | -0.32 | 0.75 |
|  |  | 10 | 1.05 (0.03) | 1.03 (0.04) | 0.24 | 0.81 |
|  |  | 11 | 0.99 (0.06) | 1.01 (0.03) | 1.12 | 0.29 |
| Thrombin time (s) | Placebo | - | 18.24 (0.88) | 18.54 (0.80) | - | - |
|  | Single-dose | 1 | 18.65 (0.92) | 17.15 (0.47) | -2.88 | 0.01 |
|  |  | 2 | 17.62 (0.88) | 17.25 (0.42) | 0.52 | 0.61 |
|  |  | 3 | 18.02 (0.81) | 17.25 (0.42) | 1.81 | 0.09 |
|  |  | 4 | 18.55 (0.69) | 18.37 (0.96) | -1.25 | 0.22 |
|  |  | 5 | 18.73 (0.87) | 18.28 (0.46) | -1.66 | 0.11 |
|  |  | 6 | 18.52 (0.64) | 18.30 (0.63) | -0.96 | 0.34 |
|  |  | 7 | 18.02 (1.30) | 17.98 (1.00) | -0.96 | 0.35 |
|  |  | 8 | 18.05 (0.56) | 18.98 (0.99) | 1.09 | 0.29 |
|  | Placebo | - | 18.37 (1.06) | 19.05 (1.58) | - | - |
|  | Multiple-dose | 9 | 18.23 (1.06) | 19.55 (2.76) | -0.08 | 0.94 |
|  |  | 10 | 19.17 (0.46) | 18.60 (0.54) | -1.45 | 0.18 |
|  |  | 11 | 18.70 (0.68) | 18.95 (0.78) | 0.40 | 0.70 |
| [Activated partial thromboplastin time](http://www.baidu.com/link?url=uTdA20YlhXBtTKZWA7CO6TJgs2dBs61yasLiPQ0202268YHZb06iTDMS55x01eZIDi4XM5evTIUIlAZYWUPqRp2kH21PqSYq2-1tmG7Yk3NNl1xEIJpalrJl9K9r8S5A20L1-3kTNHM3DXyMx1x13_) (s) | Placebo | - | 27.11 (3.48) | 26.71 (3.03) | - | - |
|  | Single-dose | 1 | 24.05 (1.92) | 25.25 (2.54) | 1.51 | 0.15 |
|  |  | 2 | 27.02 (2.32) | 25.05 (2.86) | 0.63 | 0.54 |
|  |  | 3 | 27.67 (3.71) | 25.05 (2.86) | -0.96 | 0.35 |
|  |  | 4 | 26.67 (1.15) | 27.28 (2.63) | 0.33 | 0.74 |
|  |  | 5 | 26.97 (3.27) | 27.18 (2.78) | 0.55 | 0.59 |
|  |  | 6 | 27.95 (1.58) | 27.70 (1.38) | -0.18 | 0.86 |
|  |  | 7 | 26.12 (2.45) | 25.72 (3.71) | 0.11 | 0.91 |
|  |  | 8 | 26.33 (3.63) | 26.38 (2.43) | 0.47 | 0.64 |
|  | Placebo | - | 28.23 (2.93) | 28.28 (2.13) | - | - |
|  | Multiple-dose | 9 | 26.30 (3.36) | 26.98 (1.78) | -0.64 | 0.53 |
|  |  | 10 | 30.00 (2.89) | 30.38 (4.08) | -0.08 | 0.94 |
|  |  | 11 | 27.65 (2.31) | 26.77 (0.50) | 0.16 | 0.88 |
| Fibrinogen（g/L） | Placebo | - | 2.38 (0.52) | 2.14 (0.28) | - | - |
|  | Single-dose | 1 | 2.59 (0.46) | 2.42 (0.43) | 0.28 | 0.78 |
|  |  | 2 | 2.42 (0.19) | 2.36 (0.35) | -0.41 | 0.69 |
|  |  | 3 | 2.31 (0.39) | 2.36 (0.35) | 0.88 | 0.39 |
|  |  | 4 | 2.60 (0.45) | 2.34 (0.27) | -0.26 | 0.80 |
|  |  | 5 | 2.32 (0.23) | 2.03 (0.72) | -0.18 | 0.86 |
|  |  | 6 | 2.45 (0.37) | 2.29 (0.29) | 0.04 | 0.97 |
|  |  | 7 | 2.60 (0.60) | 2.36 (0.54) | 0.37 | 0.72 |
|  |  | 8 | 2.43 (0.29) | 2.42 (0.38) | 1.28 | 0.22 |
|  | Placebo | - | 2.40 (0.53) | 2.37 (0.56) | - | - |
|  | Multiple-dose | 9 | 2.22 (0.55) | 1.94 (0.49) | -0.40 | 0.70 |
|  |  | 10 | 2.28 (0.33) | 2.18 (0.34) | -0.40 | 0.70 |
|  |  | 11 | 2.37 (0.63) | 2.19 (0.26) | -0.56 | 0.59 |

Values are mean (SD).

* Wilcoxon test of the difference between the given cohort and the placebo group in the change from baseline until after the test.
